# The Schools of Anatomy

**Published:** 1920-11-13

**Authors:** 


### THE SCHOOLS OF ANATOMY.

A short time ago Dr. Addison addressed a letter to the various Boards of Guardians pointing out that the administration of the Anatomy Acts has now been transferred to the Ministry of Health by the Home Office, as a matter deeply affecting, through the provision of properly trained medical men, the health and well-being of the country. Dr. Addison states that it will be readily appreciated that a thoroughly practical training is essential to provide the medical profession with the knowledge and skill necessary for the proper treatment of the sick and maimed. The rich can always command the services of well-trained medical men. The poor, though they cannot command, should equally be able to trust to the skill of the hands under which they are placed. The mature opinion, based on many years' experience, of the experts entrusted with the teaching of anatomy is that every medical student must do sufficient practical work to gain a knowledge of the whole complex structure of the human frame which his patients are to entrust to his care, and to acquire the delicate technique without which he should neither be expected to approach the investigation of an obscure disease, nor to dare to operate on the living.

### The Supply of Subjects.

For some time past, continues Dr. Addison, the supply of subjects for anatomical study has fallen so lamentably short of the minimum required that students of medicine have at best only limited opportunities of learning anatomy, and absolutely no chance of learning the technique of surgical operations on the dead. The result of a continued shortage in the supply must be that doctors in the future will have no alternative but to treat disease and to undertake operations in the attempt to save lives without being properly qualified to do so. The General Medical Council, which is entrusted by statute with the maintenance of an adequate standard of medical education in this country, must consent to the omission or abeyance of this fundamental training, and has already had to do so in some parts of the country.

The solution of this urgent national matter lies in the main with Boards of Guardians, who are the legal custodians of the greater number of the bodies of those dying without known relatives to claim them. If the Guardians are convinced of their public duty in this matter, and determine to co-operate to the fullest extent with the Ministry in the administration of the Acts, it is certain that, no matter what difficulties may arise in future, the needs of the medical schools can be fully met at the present time.

The antipathy felt by some to the idea of devoting unclaimed bodies to the purpose of medical education is readily understood and worthy of respect. But it has often proved on investigation to be the result of the misapprehension which has gathered round the practice of anatomy in past years; and a brief summary of the actual procedure might not be out of place, stated the Minister of Health. The body is taken from the institution to the medical school by an undertaker, on the instructions of the Inspector of Anatomy. After having been used for anatomical investigation, it is again removed in a properly made coffin by the undertaker, who completes the funeral, as in the case of an ordinary burial. The body is buried according to the rites of the religious persuasion of the deceased. A certificate of burial, signed by the chaplain and the officials of the cemetery at which the burial has taken place, is sent to the Inspector of Anatomy, who is responsible for keeping a full record of the name, age, number of grave of the deceased, and name of the clergyman who performed the burial service, as well as the date of burial. Consequently, if at any future time the friends of the deceased should make inquiries, the necessary particulars can at once be supplied.

### The Consequences.

Dr. Addison concludes by saying that an inevitable result of the relaxation of medical science will be that only students who can afford to study anatomy in foreign medical schools, a number necessarily very limited, will be competent to practise surgery without grave risk to their patients. The British medical schools, which at present compare favourably with any in the world, will be hopelessly handicapped, and, while the rich patients will be able to afford the services of foreign-trained men, those less fortunately circumstanced will suffer to a constantly increasing extent as qualified practitioners die out and are replaced by men who through no fault of their own have been unable to obtain the necessary practical training. The Minister trusted, therefore, that Guardians and other local authorities concerned will give their most earnest consideration to this question.

